# Supplementary material for: Amyloid Beta Peptides Lead to Mast Cell Activation in a Novel 3D Hydrogel Model
Source: Int J Mol Sci. 2023 Jul 26;24(15):12002. doi: 10.3390/ijms241512002 (PMC10419190; doi:10.3390/ijms241512002)
Supplement: Supplementary file 1 [file ijms-24-12002-s001.zip › ijms-2480991-supplementary.pdf]

Supplemental Figure S1

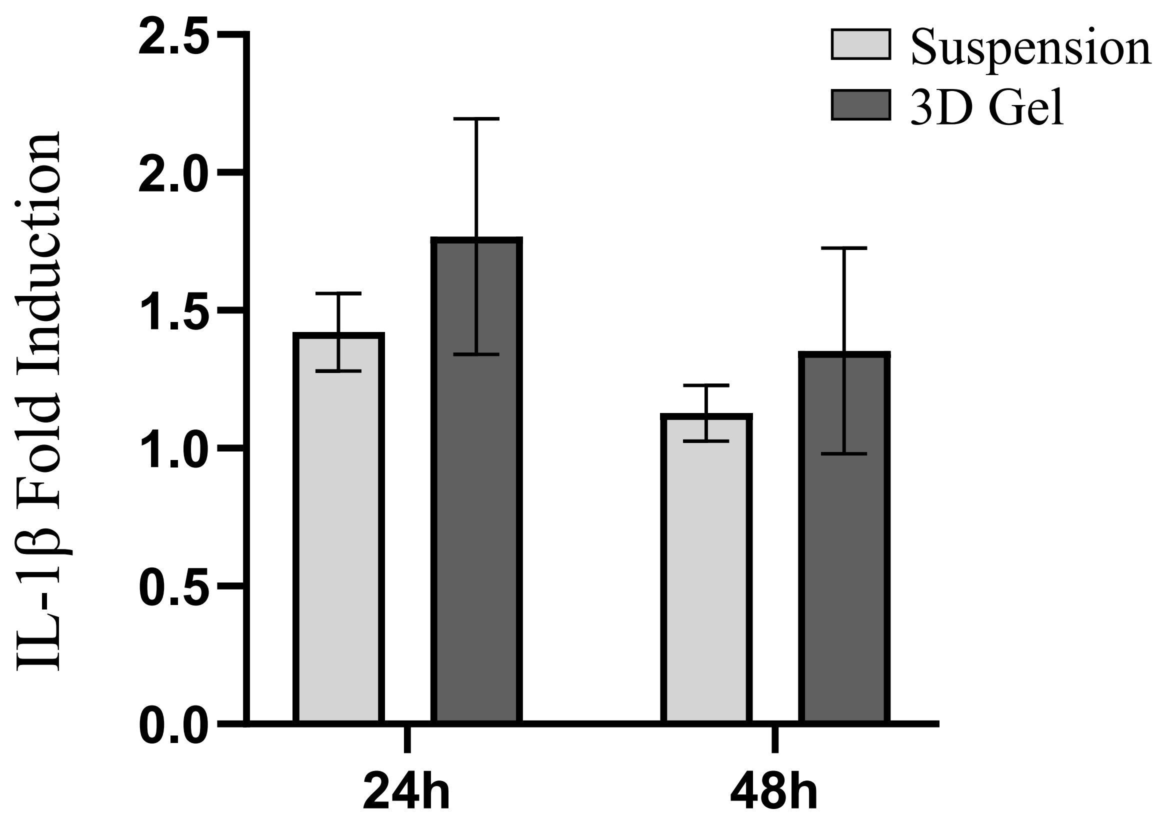

Supplemental Figure S1. Mast Cells demonstrate better response to IL-33 at 24hrs post-treatment. LADR MCs were cultured in suspension or in 3D collagen gels and treated with IL-33 (100ng/mL) for 24 or 48 hrs. ELISA results show better IL-1β fold induction at 24hrs than at 48hrs.

Supplemental Figure S2

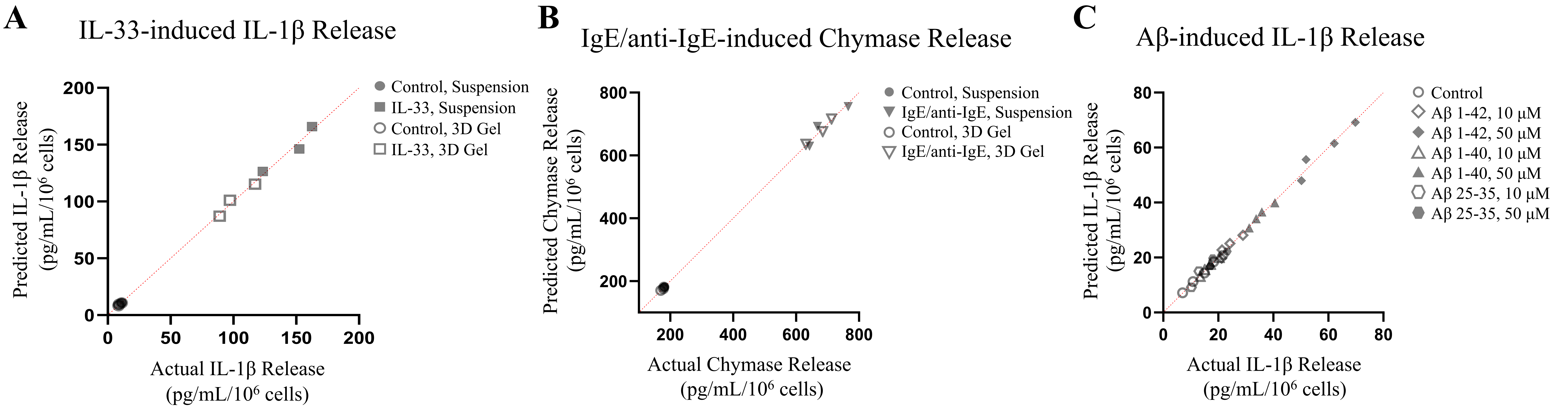

Supplemental Figure S2. Shapiro-Wilk normality test indicating normal distribution of inflammatory mediator release. ELISA results of IL-1β or chymase release from MCs were chosen. **A.** IL-33-induced IL-1β. **B.** IgE/anti-IgE-induced Chymase release. **C.** Aβ-induced IL-1β. In these QQ plots, actual IL-1β or chymase release was plotted as X axis, while predicted values based on normal distribution were plotted as Y axis. Since the actual release data is very close to the predicted distribution, it suggests that the data follows a normal distribution.
